# Preparation, characterization and *in vitro* release study of drug-loaded sodium carboxy-methylcellulose/chitosan composite sponge

**DOI:** 10.1371/journal.pone.0206275

**Published:** 2018-10-22

**Authors:** Baoyi Cai, Tian Zhong, Peizhou Chen, Jia Fu, Yuanbao Jin, Yinglei Liu, Ran Huang, Lianjiang Tan

**Affiliations:** 1 School of Pharmacy and Food Science, Zhuhai College of Jilin University, Zhuhai, Guangdong, China; 2 Department of Materials Technology and Engineering, Research Institute of Zhejiang University-Taizhou, Taizhou, Zhejiang, China; 3 School of Health, Zhuhai College of Jilin University, Zhuhai, Guangdong, China; 4 State Key Laboratory of Microbial Metabolism and School of Life Sciences and Biotechnology, Shanghai Jiao Tong University, Shanghai, China; Institute of Materials Science, GERMANY

## Abstract

A sodium carboxy-methylcellulose (CMC)/chitosan (CS) composite sponge as drug carrier was prepared, and its structure and functions were investigated. Samples with different CMC/chitosan ratios and under different pH conditions were synthesized via a freeze-drying method. The microstructure of the dried sponges was analyzed by Scanning Electron Microscope (SEM). Molecule interactions between polymers were confirmed by Fourier transform infrared (FTIR) spectra and Thermal gravimetric analyze (TGA). The swelling degree, weight loss, *in vitro* drug release behavior and antibacterial property of the sponges were determined as well. The results showed that the CMC/chitosan ratio and the pH value significantly affected the appearance of the blending solution and the microstructure of the final product, and also affected the sponge’s degradation behavior, drug-loading capacity and the antibacterial activity. Gentamicin (GEN) as a hydrophilic model drug was remarkably superior to the other two hydrophobic drugs, ibuprofen (IBU) and roxithromycin (ROX), with respect to *in vitro* releasing. Moreover, higher CMC content and lower pH value of the sponge were confirmed to lead a larger loading for GEN. The bacteriostatic experiment showed a strong antimicrobial ability of GEN-loaded sponges on inhibiting *Escherichia coli*.

## Introduction

Biodegradable sponges composed of various materials have incurred increasing interest in the last decades, and biomedical applications of such vital materials have been developed in a wide variety of fields, such as wound dressing, drug delivery and tissue engineering[1–3]. The sponge is known for a porous structure which provides potential space and channels for the containment and delivery of biofactors, subsequently it can be employed in various biomedical circumstances with the additional feature of biodegradability, which requires an assembly of raw biomaterials.

From numerous previous reports, cellulose and chitosan were determined to be excellent candidate for such integration. Cellulose is currently one of the most promising bio-based polymeric resources[4]. Due to the increasing demand for sustainable and biocompatible products, a number of functional varieties of cellulose have been developed for different biomedical applications[5]. Sodium carboxymethyl cellulose (CMC), as a representative water-soluble cellulose derivative, is manufactured by reacting sodium monochloroacetate with cellulose in alkaline. The CMC molecular structure has the monomer C_6_H_10_O_5_ linked by β-1, 4-glucosidic bonds. Moreover, CMC contains abundant hydroxyl groups so that it can easily form hydrogels with decent structures [2], which retain water molecules in a 3D gel structure, and this property contributes to tuning the flow behaviour of aqueous suspensions and solutions[6]. However, the antibacterial and mechanical properties of CMC are not very satisfactory[7].

Chitosan, as a copolymer of glucosamine and N-acetyl glucosamine unit linked by 1, 4-glucosidic bonds, is mainly obtained by N-deacetylation of chitin from shrimp or crab shells. Due to its non-toxicity, positive charge, decent mechanical properties and biocompatibility, chitosan is widely used in the biomedical area[8]. The chemical structure of the chitosan backbone is similar to that of cellulose. The abundant hydroxyl and amino groups on the backbone provide hydrophilicity and polycationic characteristics, while the amino groups can effectively inhibit the activity of bacteria by neutralizing negative charges. Moreover, the hydrophilic and high swelling properties make it possible to carry/release drugs or to culture cells[9]. Thus, as an excellent raw material, chitosan and chitosan-based composites are widely used in biomedical applications such as effective hemostatic wound dressings[10–13], bone regeneration[14] and tissue-engineering scaffolds [15–18].

Composite technology can intensify the characteristics of the initial materials [19, 20]. Previous studies have reported that composites of CMC and chitosan retain advantages from both. The chitosan molecule is well compatible with cellulose chains due to chemical similarity[21]. The stability of CMC/chitosan complex may be influenced by environmental parameters, such as pH and ionic strength, due to the protonation of amino group in chitosan and the ionization of carboxyl group in cellulose[22]. In the composite sponges, the pores were reported at the level of hundreds of micrometers [23], and the existence of chitosan in matrix provides not only anti-bacteria properties[24, 25], but also mechanical strength and structural function. Several researches have been conducted on similar drug delivery composites, for instance, chitosan/gelatin sponge for protein drug delivery[26], chitosan/polyvinyl pyrrolidone hydrogels for antibiotic delivery[27] and gelatin-alginate sponge for drug loading[28]. Based on the previous researches in this field, it can be concluded that, the physical performances and properties of the CMC/chitosan composite mainly depend on the interaction of the two kinds of polymer molecules. Moreover, the microstructure of the sponge matrix and the active chemical groups determine the drug loading capacity and the releasing behaviors.

In this work, CMC/chitosan composite sponges were prepared by freeze-drying method to investigate the influence of composition ratios and pH values on the microstructure and physical properties of the sponges. The novelty of this research is the application of a freeze-drying method to prepare the product, which has been confirmed to have certain superiority over air-drying method [29]. In the process of freeze-drying, the free water can be transformed into crystal state rapidly, and dissociated from the matrix by sublimation, which forms a porous structure in the sponge. Compared to air-drying method, the sponge obtained by freeze-drying method should have a more even porous inner structure and larger superficial area, which provide more space for drug loading with a better degradation performance. Model drugs such as Ibuprofen (IBU), roxithromycin (ROX) and gentamicin (GEN) have been applied onto our products to study the *in vitro* drug release performance and the bacteriostatic function.

## Materials and methods

### Materials

Sodium carboxymethyl cellulose (CMC, 800 ~ 1200 mPa·s of viscosity) and chitosan (CS, Mv = 5.0 × 10^5^ kDa, degree of deacetylation = 80%~95%) were purchased from Sinopharm Chemical Reagent Co. Ltd. (Shanghai, China). Ibuprofen (IBU), roxithromycin (ROX) and gentamicin (GEN) were purchased from Aladdin Chemical Co. Ltd. (Shanghai, China). O-Phthaldialdehyde (OPA) was purchased from Kuer bio-engineering Co. Ltd. (Shanghai, China). The 2-mercaptoethanole was purchased from Vorradex Reagent Co. Ltd. (Shanghai, China). *Escherichia coli* were supplied by China Center of Industrial Culture Collection (CICC 10389). Other chemical reagents used for following investigations were of analytical grade.

### Preparation of blend solutions

The two solutions with appropriate concentrations were produced by dissolving chitosan and CMC powder in 1 wt% acetic acid and distilled water, respectively. In order to avoid the dough formation, CMC and chitosan powders were added slowly into the solution with constant magnetic stirring for 1 hour at ambient temperature. After the contents were completely dissolved, the pH value of both solutions was adjusted to 1 with hydrochloric acid. Then, the two solutions were blended together and placed in the fuming cupboard for 12 h to remove the acetic acid. At last, pH of blending solution was adjusted to a certain value by hydrochloric acid or sodium hydroxide. The detailed ratios of the samples were listed in Table 1.

**Table 1:** The parameters (ratios and pH values) and viscosities of CMC/chitosan blend solutions.

Sample	Mixing ratio		pH value	Viscosity (Pa·s)
	CMC	Chitosan		
CS	0	10	-	0.0205(0)^c^
CCS28	2	8	-	1.763(0.9453)^c^
CCS46	4	6	-	62.7217(2.108)^a^
CCS64	6	4	-	63.6457(0.9818)^a^
CCS82	8	2	-	14.4907(5.4489)^b^
CMC	10	0	-	0.2749(0.0004)^c^
CCS46-pH1	4	6	1	0.0268(0)^c^
CCS46-pH5	4	6	5	32.564(2.933)^b^
CCS46-pH9	4	6	9	47.1947(4.7043)^a^
CCS46-pH13	4	6	13	0.0705(0.0001)^c^

Data reported are mean values and standard deviation (in parenthesis).

^a, b, c^ Different superscripts within a column indicate significant differences among samples (*p* < 0.05).

### Preparation of OPA reagent

OPA reagent was prepared by the following procedure[30]: 200 mg of o-phthaldialdehyde was dissolved completely in 2 mL of methanol under ultrasonic treatment. The solution was mixed with 38 mL of a 0.4 M boric buffer and the pH was adjusted to 10.4. Then, 0.4 mL of 2-mercaptoethanole was added into and potassium hydroxide was used for adjusted the pH value to be 10.4. Because OPA is unstable in aqueous solution[31], the OPA reagent was stored at 4°C for 48 h ~ 72 h. Sample derivatization was conducted by adding 1000 μL release media and 1500 μL OPA reagent, and heated in water bath for 20 min. The OPA reagent reacted with amino groups to form the GEN and chromophoric products, which can be detected in UV spectra.

### Preparation of sponge

All the CMC/chitosan composites were placed in the refrigerator at -18°C for 12 h for pre-freeze treatment. Then the samples were freeze-dried for 24 hours to eliminate the solvent. Sponge samples obtained were then stored in a vacuum drying oven for the following experimental characterizations.

### Solution viscosity test

The viscosity of the composite solutions was analyzed with a digital rotating viscosity meter (SNB-2, Jingtian, Shanghai, China) in triplicate at room temperature.

### Morphology of the sponge

The morphology of sponge refers to macro indicators including color, surface feature and the level of uniformity and flexibility. During the test, sample CCS46-pH1 was folded in half and then released. The initial, folded and recovered states of the sponge were photo-recorded.

Microstructure analysis of the sponges was carried out by observing both the surface and cross-section micrographs of the scanning electron microscopy (ZEISS SUPRA 55, SEM, Germany). After maintaining in a desiccator for 48 h, the sponge samples for surface observation were cut into 3 mm × 3 mm slices for SEM observation, while some samples were frozen in liquid N_2_ and broken into small pieces to observe the cross-section.

### Fourier transform infrared spectroscopy (FTIR) and Thermal gravimetric analyze (TGA)

The FTIR spectra was carried on IR Prestige-21 (Shimadzu, Japan). The TGA was carried on DTG-60H (Shimadzu, Japan) with heating speed of 10°C/min from 30°C to 600°C under nitrogen atmosphere. The samples were pre-heated to 100°C to remove the absorbed water and cooled down to ambient temperature for the measurements.

### Swelling and weight loss test

In order to test the swelling/weight loss, the sponges were pre-prepared by firstly dried for six hours in vacuum to remove extra moisture. Phosphate Buffered Saline (PBS) was precisely prepared and adjusted pH value to 7.4 by NaH_2_PO_4_ and Na_2_HPO_4_.

Each pre-weighted sample sponge (approximately 2×2 cm^2^ in size) was completely immersed in 20 mL of PBS. At specific time intervals (30 min, 60 min, 90 min, 120 min, 180 min and 12 h), the samples were drained from PBS and blotted with filter paper to remove extra water on the surface. The increased weight of wet samples, which represented the water absorbed by the sponges in aqueous environment, was calculated. After 12 hours of immersion, the sponges were dried and the weight was recorded for the weight loss calculation. The Eqs (1) and (2) were used to determine the swelling degree (*SD*) and weight loss (*WL*) of the sample, respectively.

$$SD=100\;(W_{s}‑W_{i})/W_{i}\;(\%),$$ (1)

where *SD* is the swelling degree of the sponge, *W*_*s*_ donates the weight of the sponge at specific time, while *W*_*i*_ is the initial weight of the sponge;

$$WL=100\;(W_{i}‑W_{d})/W_{i}\;(\%),$$ (2)

where *WL* represented weight loss and *W*_*i*_ and *W*_*d*_ are the weights of the initial dried samples, and the dried sample which had been immersed in media for 12 h.

### *In vitro* release

Ibuprofen (IBU) and roxithromicin (ROX) were chosen as the model hydrophobic drug and gentamicin (GEN) as hydrophilic. Sample sponges (weight about 0.25 g of each) were immersed respectively in 1% (w/w) IBU, ROX and GEN solutions. After 48 hours’ standing in an incubator at 37°C, the drug loaded samples were quickly washed by PBS and gently blotted with filter papers to remove the surface water, and then placed in a vacuum drying oven for 24 hours. Referring to the method reported before[32], drug release behavior was conducted with 20 mL PBS (pH 7.4, 37°C). Drug release media (1 mL) was withdrawn at particular time intervals (5 min, 15 min, 30 min, 60 min, 90 min, 120 min, 150 min, 180 min and 12 h) and replaced by fresh PBS of same volume. Each withdrawn sample was tested by UV spectra (Shimadzu UV-VIS 2450, Japan). Sample data collection was optimized with UV detection at 236 nm for IBU, 202 nm for ROX and 337 nm for GEN. Analysis of GEN obeyed the OPA assay because of the poorly absorption of ultraviolet and visible light, and the lack of fluorophores[30, 31]. The data of concentration was added to the cumulative mass of these drugs that had already been removed from each 1mL release media.

### Antibacterial properties

Antibacterial activities of pH group samples were assessed. Before the test, the sponges were cut into small pieces (7 mm in diameter). Samples were immersed in 1% gentamicin solution to load antibiotic and then dried in vacuum oven to remove moisture, while the control samples without gentamicin solution treatment were also prepared for comparison. The standard microbes used in this test were *Escherichia coli*. Under an asepsis operation condition[13], each sample piece was placed in the center of the culture dish and then were incubated overnight at 37°C. After 24 hours, the diameters of clear circle zone were measured and recorded for analysis.

### Statistical analysis

Results were reported as the mean of at least three measurements from three different preparations. Statistical differences in the sample properties were determined by one-way analysis of variance (ANOVA) and Duncan’s multiple range test (*p* = 0.05).

## Results and discussion

### Solution viscosity test

**Fig 1:**
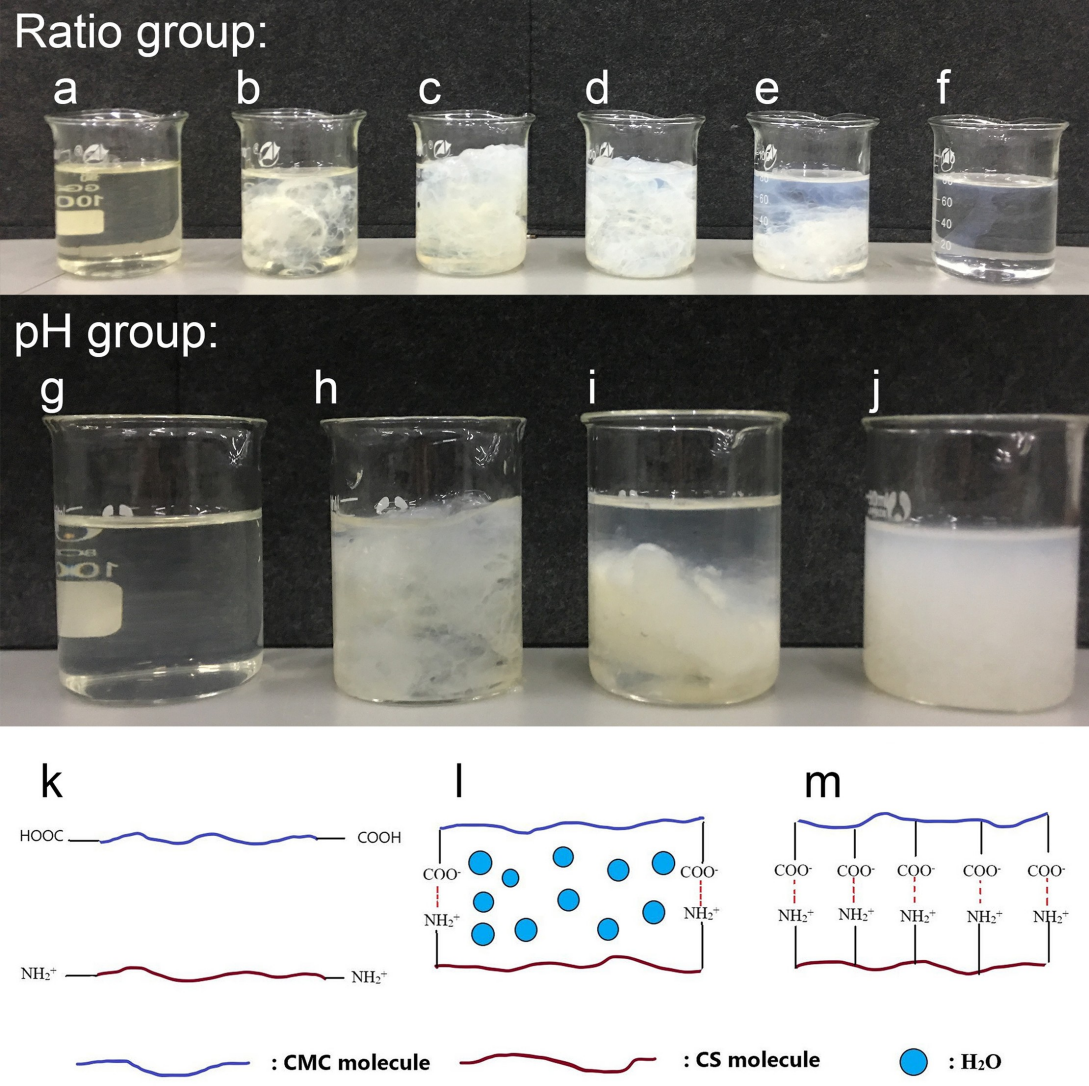
**Photographs of CMC/chitosan blend solutions (gel) under different pH conditions and the Schematic diagrams of the interaction between CMC and chitosan molecules: photographs of ratio group.** (a) chitosan, (b) CS28, (c) CS46, (d) CS64, (e) CS82, (f) CMC; photographs of pH group (g) CCS46-pH1, (h) CCS46-pH5, (i) CCS46-pH9, (j) CCS46-pH13; Schematic diagrams: (k) CCS46-pH1, (l) CCS46-pH5 and CCS46-pH9, (m) CCS46-pH13.

The viscosities of both composite ratio and pH group were tested and the result is shown in Table 1. For the ratio group, CCS46 and CCS64 presented notably higher values than other samples. Referring to Fig 1A–1F, a flocculating substance, which is isoelectric, appeared when the two kinds of polymers mixed. The flocculating substance twisted around the rotor of the viscometer and caused a dramatic increase in viscosity.

For the pH group, the viscosities of CCS46-pH5 and CCS46-pH9 are much higher than CCS46-pH1 and CCS46-pH13, according well to the appearance of blending samples shown in Fig 1G–1J. With the addition of H^+^, the flocculating substance vanished gradually and the transparent solutions were recovered (Fig 1G). In this case, H^+^ destroyed the hydrogen bonds between carboxyl groups in CMC and hydroxyl groups in chitosan, and the polymer chains separated from each other (Fig 1K). In the opposite direction, with the addition of–OH, the flocculating substance transformed into sediment particles suspended in the media (Fig 1J). As illustrated in Fig 1M, without free hydrogen ions, two kinds of polymers combined closely via ionic bonding, and there was no extra space available to retain water, consequently the polymer molecules associated together into large particles, which finally produced a low-transparency suspension with low viscosity. A similar result was obtained in a previous work concerned the chitosan-alginate complex[22].

### Morphology of the sponge

**Fig 2:**
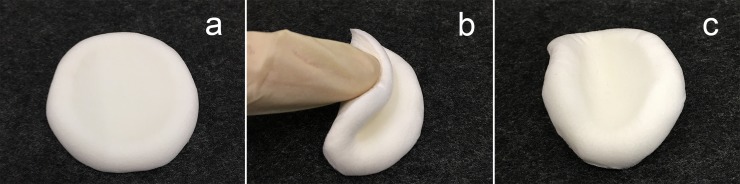
**Morphology of the composite sponge.** (a) flattened sponge, (b) folded sponge, (c) recovered sponge.

A dry CMC/chitosan composite sponge (CCS46-pH1) prepared by freeze-drying is shown in Fig 2. The white, uniform, and porous surface implies that a good dispersion of the components was achieved with a compact and homogeneous structure. The composite sponge was soft enough to be easily folded at room temperature. When the pressure was released, the sponge rebounded from the deformation to the initial shape gradually, which suggests a good flexibility and elasticity.

**Fig 3:**
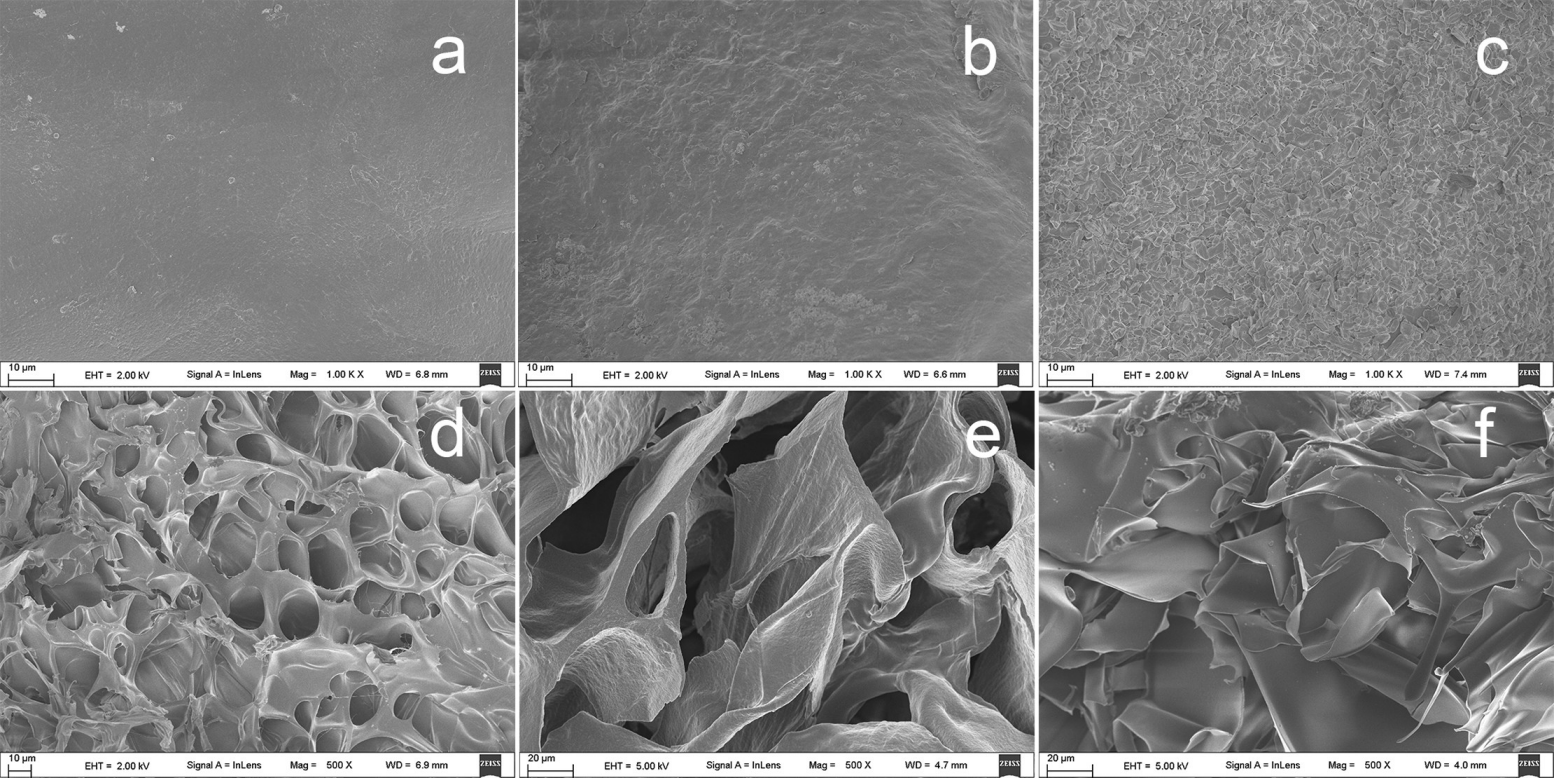
**SEM photographs of the composite sponges.** Surface: (a) CCS46-pH1, (b) CCS46-pH5, (c) CCS46-pH13; cross-section: (d) CCS46-pH1, (e) CCS46-pH5, (f) CCS46-pH13.

SEM graphs of the CMC/chitosan composite sponges are shown in Fig 3. The CCS46-pH1 (Fig 3A) exhibited the most compact and uniform surface morphology among these samples. This implies that a solution with a more homogeneous state more easily forms a uniform and intact surface after freeze-drying. As the pH value rises, a more rough and irregular surface can be observed in sample CCS46-pH5 (Fig 3B) and CCS46-pH13 (Fig 3C). It is probably due to the absence of the influence of hydrogen ions that the strong intermolecular hydrogen bonds between CMC and chitosan might form a chaotic gel state in the solution, which leads to a rough surface.

In contrast, the cross-section SEM graphs of the three samples all present a large, open, channel-like structure with different size pores. Compared to the other two samples, CCS46-pH5 (Fig 3E) presents the largest average pore size, which is due to the CCS46-pH5 holding the most water in its hydrogel matrix (Fig 1B) before freeze-drying, and subsequently during the freeze-drying process, large blocks of ice sublimated, and left the irregular pores inside the dried sponges. For the CCS46-pH1 (Fig 3D), the high hydrogen (H^+^) concentration dissociated the two polymer chains, which resulted in a uniform distribution of polymer chains and water particles. In Fig 3F, a chaotic layered structure can be observed in the cross-section of CCS46-pH13. According to the mechanism demonstrated in Fig 1G, the polymer chains associated together closely due to the high pH and there was less space to hold water molecules, thereafter it lacked of the regular three-dimensional gel structure which can effectively support the sponge morphology.

### Fourier transform infrared spectroscopy (FTIR)

**Fig 4:**
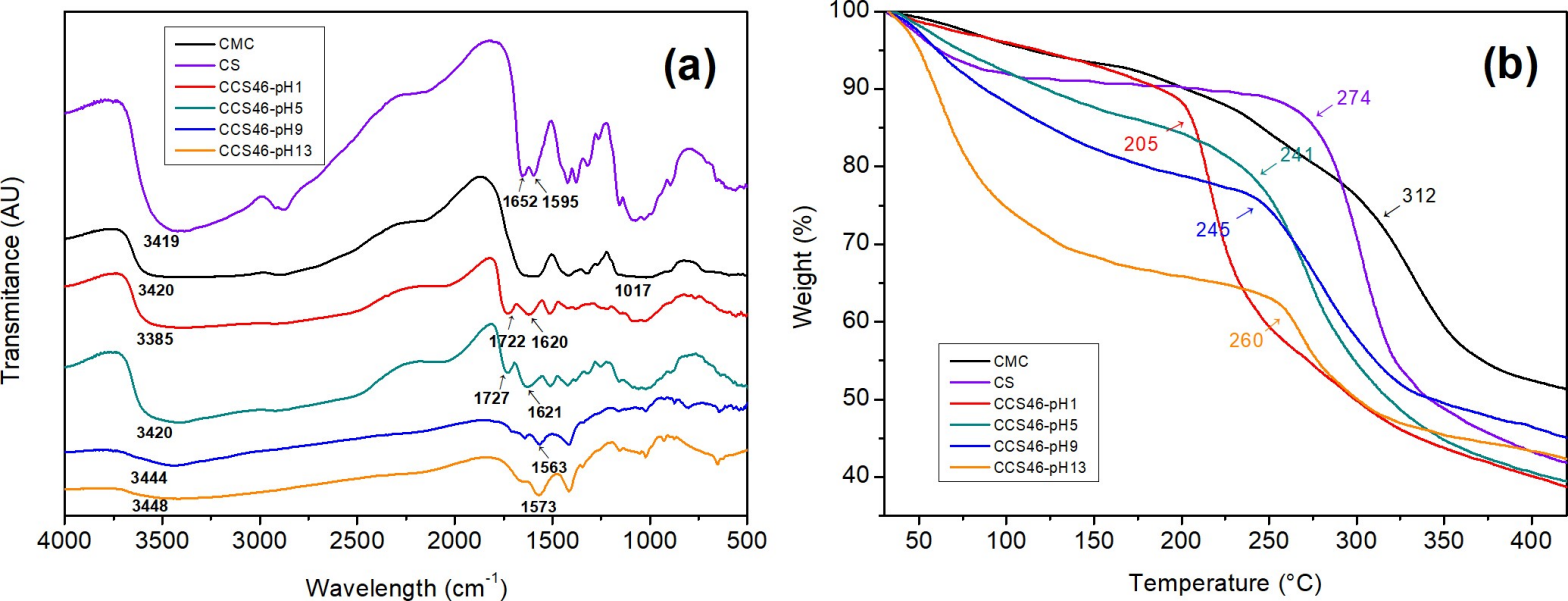
FT-IR spectra (a) and TGA spectra (b) of the pH group samples.

The FTIR spectra of the sponges are shown in Fig 4A. The peaks around 897 cm^-1^ and 1160 cm^-1^ in the spectra refer to the saccharide structure[23], and the ether C–O–C functional groups are found around 1017.35 cm^-1^ for CMC. The two peaks at 1652.15 cm^-1^ and 1595.78 cm^-1^ in the chitosan spectrum refer to the amide-I and amide-II respectively. A broad band at 3450 cm^-1^ ~ 3380 cm^-1^ is the signal of–OH stretching vibrations and–NH–stretch[2]. In the composite sponges, the–NH–stretching vibration band locates from about 3448 cm^-1^ to 3385 cm^-1^. The similarity of composite and single components can confirm that the sponges have good compatibility and miscibility [33]. For CCS46-pH1 and CCS46-pH5, the absorption peaks of the C = O band are located at 1722 cm^-1^ and 1727 cm^-1^, and the peaks of the–NH groups were at 1620^−1^ and 1621^−1^, respectively. However, for CCS46-pH9 and CCS46-pH13, the peaks of C = O shifted to a lower frequency and vanished gradually. This phenomenon indicated that in the relatively higher pH samples, the CMC and chitosan molecules combined more closely by hydrogen bonding, which was in accordance with Fig 1M. In addition, the peak around 3420 cm^-1^ for–OH or–NH groups shifted from 3385 cm^-1^ (CCS46-pH1) to 3448 cm^-1^ (CCS46-pH13), with the increase of band width. It indicated the formation of new intermolecular hydrogen bonds between the two polymers.

### Thermal gravimetric analyze (TGA)

The thermal stability and thermal decomposition of the pH group samples were investigated by TGA analysis and are presented in Fig 4B. From the initial temperature of 30°C to 200°C, samples did not decompose. In this region, free water was still contained in the sponges, and was the main factor of the initial weight loss of sponges below 200°C.

The initial degradation temperatures stand at about 312°C for CMC and 274°C for chitosan. For the pH group (CCS46-pH1, CCS46-pH5, CCS46-pH9 and CCS46-pH13), the initial degradation temperatures were shifted to a lower temperature range (205 ~ 260°C). It is known that there is no melting transition in CMC and chitosan because of the hydrogen bonds, and in the composite the interactions between the two polymers effectively weaken the intra-and intermolecular hydrogen bonds[33]. In addition, the initial degradation temperature dropped as the pH descended. CCS46-pH1 showed the lowest initial degradation temperature (205°C) among the samples. It is reasonable to hypothesize that at the condition of low pH value, the intra-and intermolecular hydrogen bonds were weakened by the abundant H^+^ ionic, and the structure became easier to be decomposed under heat. This test proved the existence and the significant effect of the hydrogen bonds in the composite matrix again, which is also the main reason of the viscosity increasing and the band changing in previous viscosity and FT-IR tests.

### Swelling/weight loss test

**Fig 5:**
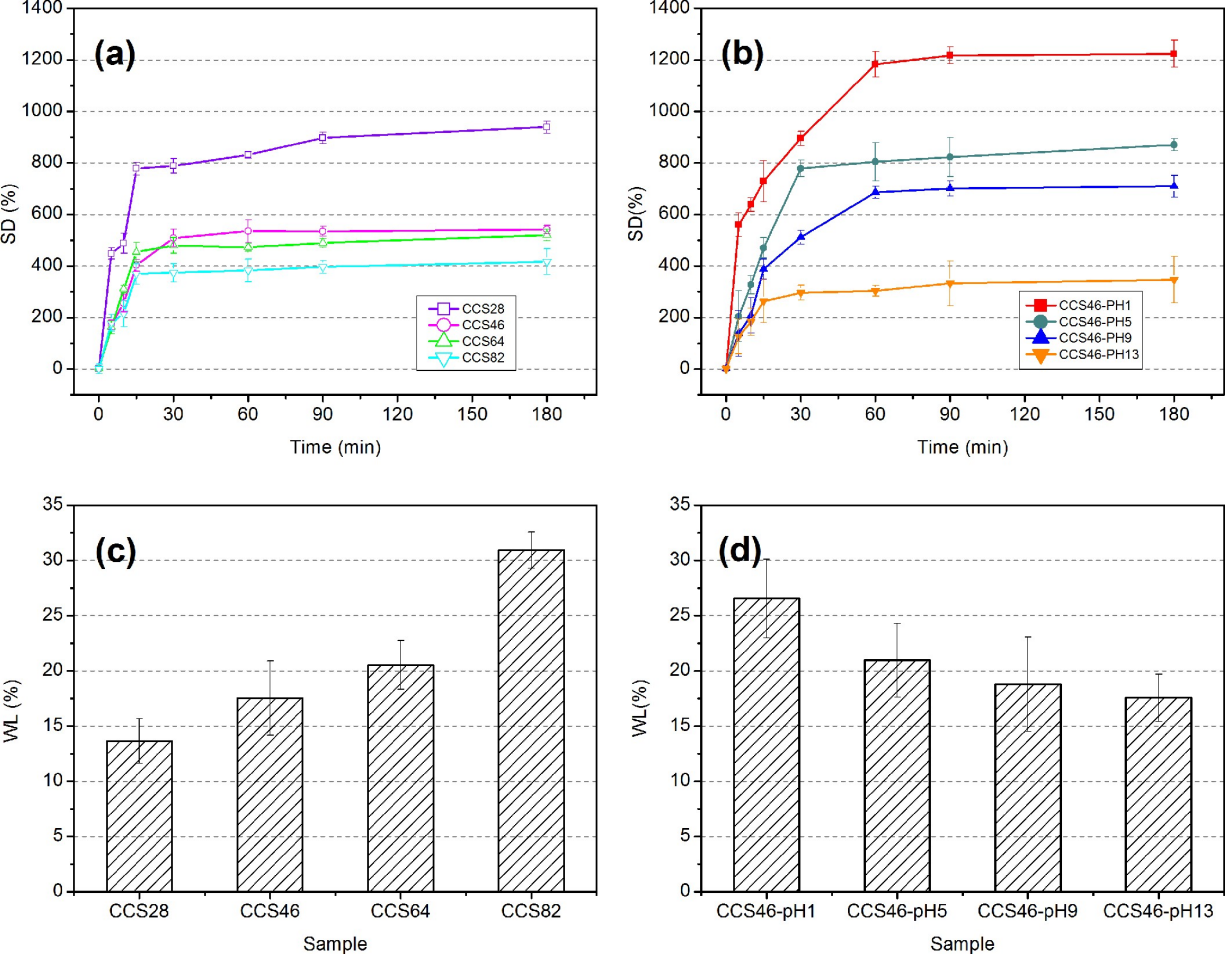
**Swelling degree (SD) and weight loss (WL) of the samples.** (a) SD of ratio group, (b) SD of pH group, (c): WL of ratio group, (d): WL of pH group.

The effects of component ratios on swelling degree (SD) and weight loss (WL) were tested and the results are given in Fig 5. For the ratio group (Fig 5A), all the sponges swell fast at the beginning 15 min and the curves stabilize after 30 min. It can be observed that the swelling degree increases as the CMC content decreases. CCS28 with only 20% of CMC proportion shows the best swelling ability, which absorbed 939.33% (w/w) of water, while CCS82 with 80% CMC shows the worst value (417%) of water absorption. This showed that the CS molecules show better extensibility than CMC, thus the lower CMC proportion sample presents a better swelling ability in the water. In addition, CCS46 and CCS64 presented a relatively poor capability of swelling. Combined with the previous results of viscosity and FT-IR test, it could be inferred that because of more interaction between the two molecules inside, the materials made of a balanced CMC/CS ratio can hardly swell to hold water. For the pH group (Fig 5B), a regular relationship can be found between pH value and swelling degree, and the swelling degree declines as the pH rises. Sample CCS46-pH1 presents the maximum value (1224%, w/w) of swelling degree, while CCS46-pH13 presents the lowest (347%, w/w). This is because the highly cross-linked composite network in the higher pH sample has less space to contain water molecules, so the sponge cannot absorb too much water and retain it inside.

The results of the weight loss present a regular increase as the CMC content increases in ratio group (Fig 5C) and a decline as the pH value increases in pH group (Fig 5D). For the ratio group, the higher the CMC content, the more quantity the composite matrix would lose. The sample CCS82 shows the maximum value which is over 30% (w/w). The main reason is the hydrophilic nature of CMC, which results in a good dissolution in the aqueous environment. Nevertheless, the chitosan retains some portion of CMC, by the interaction between amino and carboxyl groups. For the pH group (Fig 5D), the weight loss drops gradually as the pH value rises. It is because that, when the sponges swelled, the amorphous chains have more freedom to move, thus the CMC in the matrix can easily detach from the network and dissolves into the media. Thus, as the swell degree goes down, it becomes harder for the CMC molecules to dissolve. In brief, higher CMC proportion and lower pH value lead to a better ability of degradation in the CMC/chitosan composite sponges.

### *In vitro* release study

**Fig 6:**
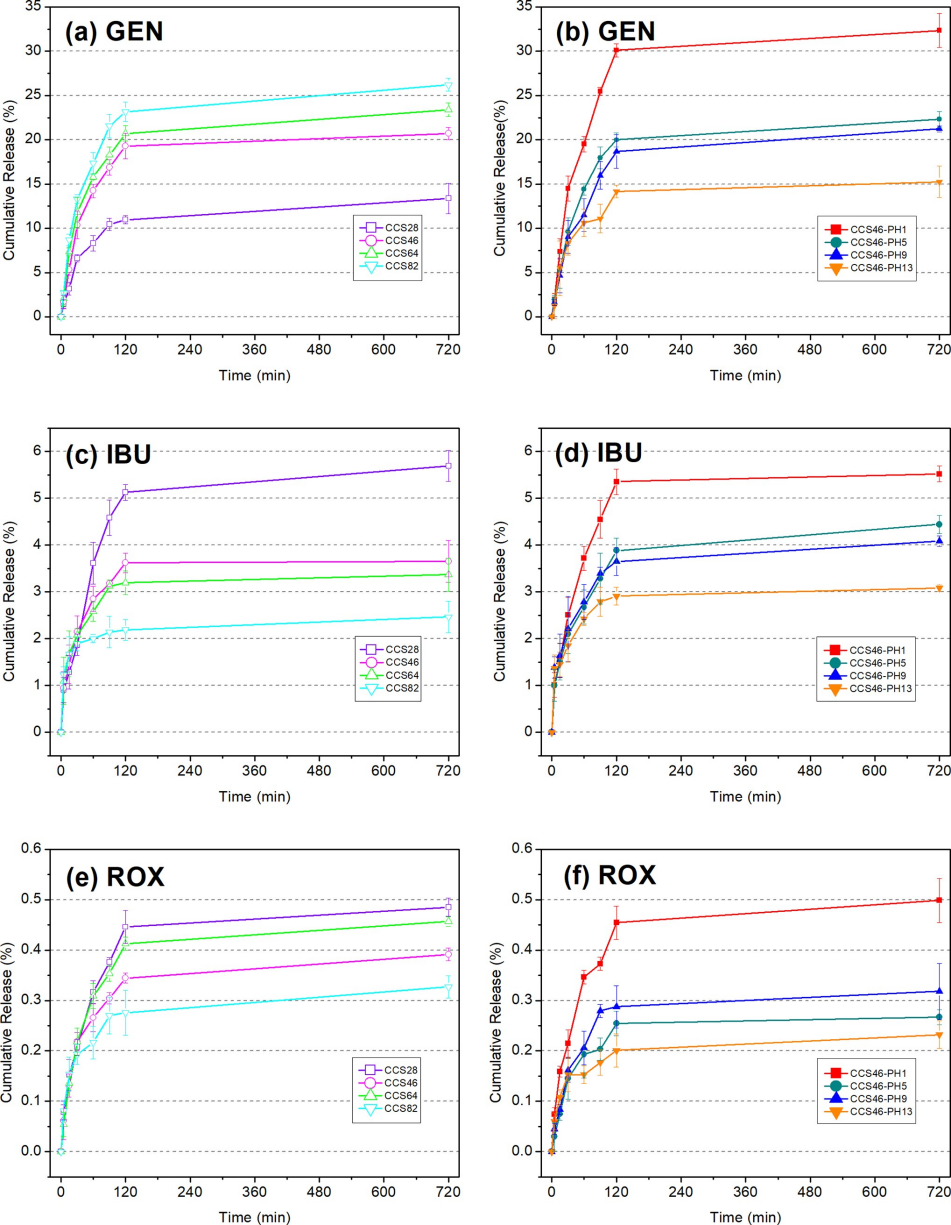
**Drug cumulative release of sponges.** (a) GEN of ratio group, (b) GEN of pH group (c) IBU of ratio group, (d) IBU of pH group, (e) ROX of ratio group, (f) ROX of pH group.

Fig 6 shows the drug release behavior of sponges which were prepared with different ratios and pH values. Initial burst release was observed in the first 60 min for each sample, and the release became stable after 120 min. This phenomenon is in accordance with the behavior of swelling degree. The good solubility of CMC in PBS also contributed to the degradation of the composite sponge matrix, which accelerated release rate during the initial burst. In this study, *in vitro* release of gentamicin (GEN) was compared to that of ibuprofen (IBU) and roxithromycin (ROX). The maximum value of the hydrophilic GEN’s cumulative release was 32.36%, which was remarkably higher than the two other hydrophobic drugs (5.69% for IBU and 0.5% for ROX). This is primarily due to the interactions between GEN’s amino groups and CMC’s carboxyl groups[28], which provided the composite sponge an excellent GEN loading capacity. According to the drug release curves of the ratio group (Fig 6A, 6C and 6E), the composition ratio of the sponges tended to be a significant factor to affect the release behavior. In the GEN’s curves (Fig 6A), the cumulative release rate is in proportional to the CMC content, and the CCS82 sample which has 80% (w/w) of CMC performs the best with respect to drug loading ability. In contrast, no special interaction was found between the sponge and the two hydrophobic drugs. Instead, the sponge swelling is the main driving force of the IBU and ROX’s releasing. For this reason, the cumulative release rate curves of IBU and ROX showed an opposite tendency to that of GEN. For the pH group (Fig 6B, 6D and 6F), an identical tendency was observed in the curves of all three drugs. The cumulative release decreases as the pH value increases, and the CCS46-pH1 sample presents the best performance of loading and releasing out of all three drugs. This could also be explained by the relationship between drug releasing and sponge swelling. Additionally, for GEN, the large amounts of H^+^ in the solution made the carboxyl groups of CMC detached from the amino groups of chitosan molecules and become exposed, which provides a large number of vacancies for the amino groups of GEN molecules.

### Antibacterial properties

**Fig 7:**
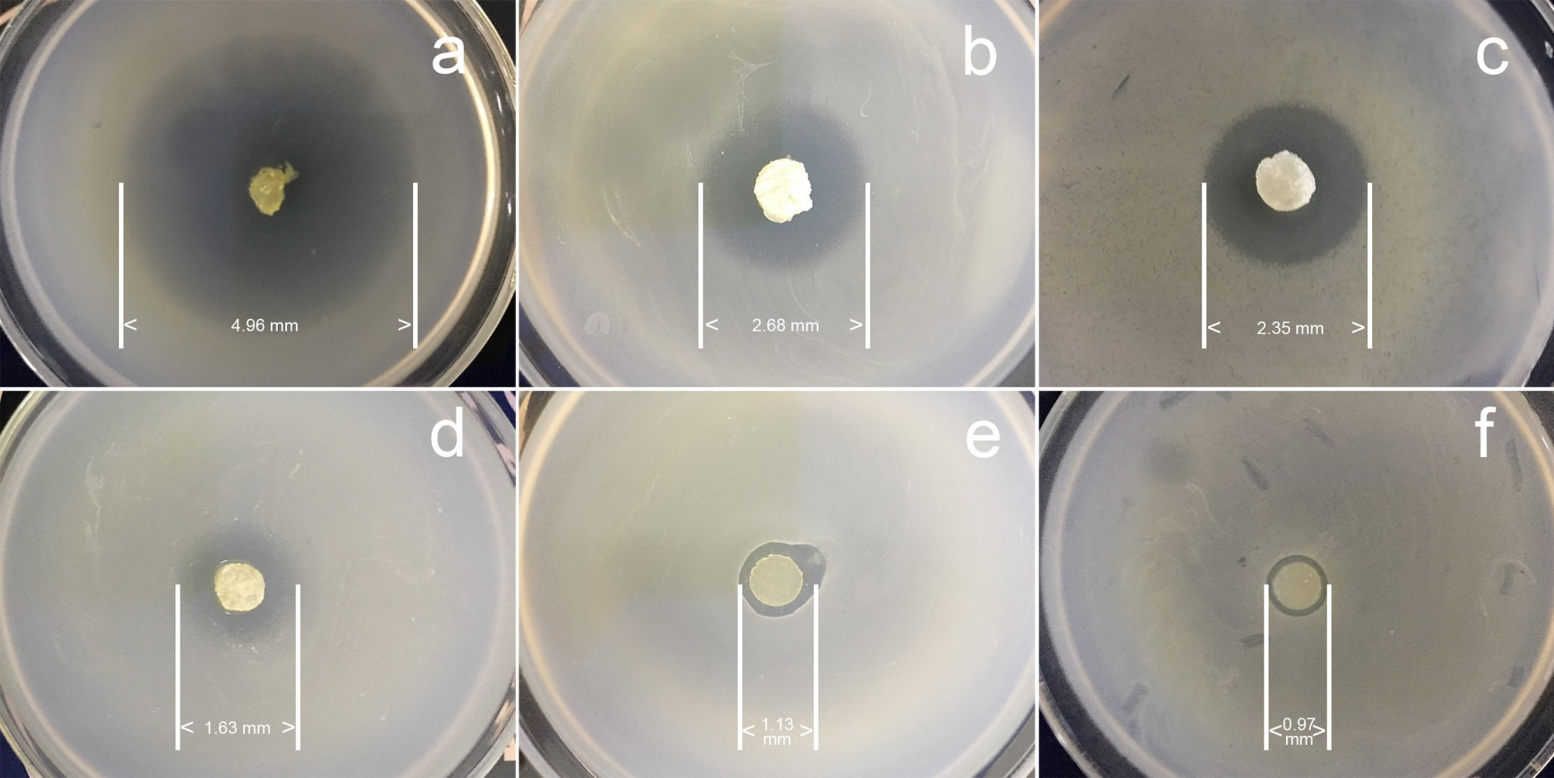
**Antibiotic circles of samples loading GEN and control samples.** GEN group: (a) CCS46-pH1, (b) CCS46-pH5, (c) CCS46-pH9; Control group: (d) CCS46-pH1, (e) CCS46-pH5, (f) CCS46-pH9.

The sizes of zones of inhibition of the sponges were shown in Fig 7. From the photos we could conclude that all the sponge samples have the ability to restrain the growth of *Escherichia coli* to a certain degree. For the GEN group (Fig 7A, 7B and 7C), the diameters of the antibacterial circles are 4.96 mm (CCS46-pH1), 2.68 mm (CCS46-pH5) and 2.35 mm (CCS46-pH9). In contrast, the control group (Fig 7D, 7E and 7F) presented the observations of 1.63 mm (CCS46-pH1), 1.13 mm (CCS46-pH5) and 0.97 mm (CCS46-pH9) in diameter. This obviously illustrates the superiority of the GEN loading samples over the control ones on inhibiting *Escherichia coli* K88. Referring to studies on the drug release behavior shown in Fig 6B, various pH value induced a different cumulative release curve, and the same mechanism made the sample CCS46-pH1 have a significantly better GEN loading capacity than CCS46-pH5 and CCS46-pH9. On the other hand, the antibacterial property of the control samples was mainly attributed to chitosan, which has the natural antibacterial activity as a result of its ability to neutralize negative electrons in bacteria with radical amino groups (-NH_2_) of positive charge. The control group samples (Fig 7D, 7E and 7F) CCS46-pH1, CCS46-pH5 and CCS46-pH9 presented a gradient attenuation in diameters, which is due to the decreasing dissociating amino groups and corresponding natural antibacterial activity of chitosan.

## Conclusion

In this work, CMC/chitosan composite sponges as drug carriers were successfully prepared by freeze drying process. According to the observation on camera photos and SEM, a relatively higher ratio produced more hydrogel in the blending solution, which results in a more uneven and chaotic microstructure of the final sponge. In addition, the low pH sample presented a more uniform distribution and dimensional regularity than the high pH sample. The FTIR results confirmed the formation of intermolecular hydrogen bonds between the two polymers, especially in the high pH samples. In the TGA spectra, the composite samples showed an overall decline on the initial degradation temperature compared with the pure CMC and chitosan. However, with an increasing pH, the initial degradation temperature shifts to a higher range due to the intra-and intermolecular interactions. The sponge CCS46-pH1 (pH = 1) presented the best performance (1224%, w/w) on swelling degree. The increase of the CMC content and the pH value both bring down the swelling degree. For the weight loss test, the CMC content mainly affected the degradation due to the CMC’s natural solubility in water. Besides, the samples made in low pH led to a more notable degradation of the sponge. The *in vitro* drug-releasing test showed that the optimum value of cumulative release of the hydrophilic drug GEN reached 32.36% (w/w), which is remarkably superior to the other two hydrophobic drugs (IBU and ROX). The higher CMC content and lower pH value of the sponge led to a larger GEN loading. In bacteriostatic experiment, limited antibacterial property of the control sponges without drug was confirmed due to the existence of chitosan, while the GEN-loaded sponges have a more remarkable effect on inhibiting *Escherichia coli*.

## Supporting information

S1 Table Raw data of FT-IR spectra, TGA spectra, swelling degree, weight loss and drug cumulative release of sponges. (XLSX)
